# A Contemporary Review of the Effects of Exercise Training on Cardiac Structure and Function and Cardiovascular Risk Profile: Insights From Imaging

**DOI:** 10.3389/fcvm.2022.753652

**Published:** 2022-02-21

**Authors:** Waleed Alhumaid, Stephanie D. Small, Amy A. Kirkham, Harald Becher, Edith Pituskin, Carla M. Prado, Richard B. Thompson, Mark J. Haykowsky, D. Ian Paterson

**Affiliations:** ^1^Division of Cardiology, Mazankowski Alberta Heart Institute, University of Alberta, Edmonton, AB, Canada; ^2^Faculty of Kinesiology, University of Toronto, Toronto, ON, Canada; ^3^Faculty of Nursing, College of Health Sciences, University of Alberta, Edmonton, AB, Canada; ^4^Department of Agricultural, Food and Nutritional Science, University of Alberta, Edmonton, AB, Canada; ^5^Department of Biomedical Engineering, University of Alberta, Edmonton, AB, Canada

**Keywords:** cardiovascular disease, exercise training, imaging, left ventricular function, vascular function, body composition

## Abstract

Exercise is a commonly prescribed therapy for patients with established cardiovascular disease or those at high risk for *de novo* disease. Exercise-based, multidisciplinary programs have been associated with improved clinical outcomes post myocardial infarction and is now recommended for patients with cancer at elevated risk for cardiovascular complications. Imaging studies have documented numerous beneficial effects of exercise on cardiac structure and function, vascular function and more recently on the cardiovascular risk profile. In this contemporary review, we will discuss the effects of exercise training on imaging-derived cardiovascular outcomes. For cardiac imaging *via* echocardiography or magnetic resonance, we will review the effects of exercise on left ventricular function and remodeling in patients with established or at risk for cardiac disease (myocardial infarction, heart failure, cancer survivors), and the potential utility of exercise stress to assess cardiac reserve. Exercise training also has salient effects on vascular function and health including the attenuation of age-associated arterial stiffness and thickening as assessed by Doppler ultrasound. Finally, we will review recent data on the relationship between exercise training and regional adipose tissue deposition, an emerging marker of cardiovascular risk. Imaging provides comprehensive and accurate quantification of cardiac, vascular and cardiometabolic health, and may allow refinement of risk stratification in select patient populations. Future studies are needed to evaluate the clinical utility of novel imaging metrics following exercise training.

## Introduction

Regular physical exercise provides many benefits to the cardiovascular system and overall health at all stages of life. As such, aerobic exercise (e.g., walking, cycling) and more recently resistance exercise (e.g., weightlifting), have been an integral component of clinical guidelines, and the cornerstone of cardiac rehabilitation for patients with or at risk for cardiovascular disease including coronary artery disease (CAD), heart failure, and cancer (1–4).

The most common form of exercise for primary and secondary prevention of cardiovascular disease is aerobic training, which improves oxygen delivery (i.e. cardiac output) during physical effort, and resistance training, which increases skeletal muscle mass and strength. Patients with established cardiovascular disease are commonly referred to cardiac rehabilitation, which typically involves a 6–24-week program of supervised, moderate-intensity (50–75% of maximal heart rate or 40–60% of heart rate reserve) continuous aerobic exercise, supplemental resistance training and other health interventions. High-intensity interval training (brief periods of 85–100% maximal heart rate or 80–90% heart rate reserve alternated with rest or low intensity) is a time efficient aerobic exercise alternative for lower risk patients and appears to offer similar health benefits (5). Exercise training interventions have been shown to exert direct and indirect beneficial effects on the cardiovascular risk profile. Aerobic and resistance training prevent or reduce insulin resistance and diabetes mellitus type II (6), and have favorable effects on blood pressure, lipid profile, vascular inflammation, body composition, and overall cardiac function in patients with established cardiac disease as well as healthy individuals (7–9). Dietary counseling for optimal nutritional study also plays an integral role in most rehabilitation programs and targeted nutrition interventions can improve body composition, metabolic and cardiovascular health (10).

**Figure 1:**
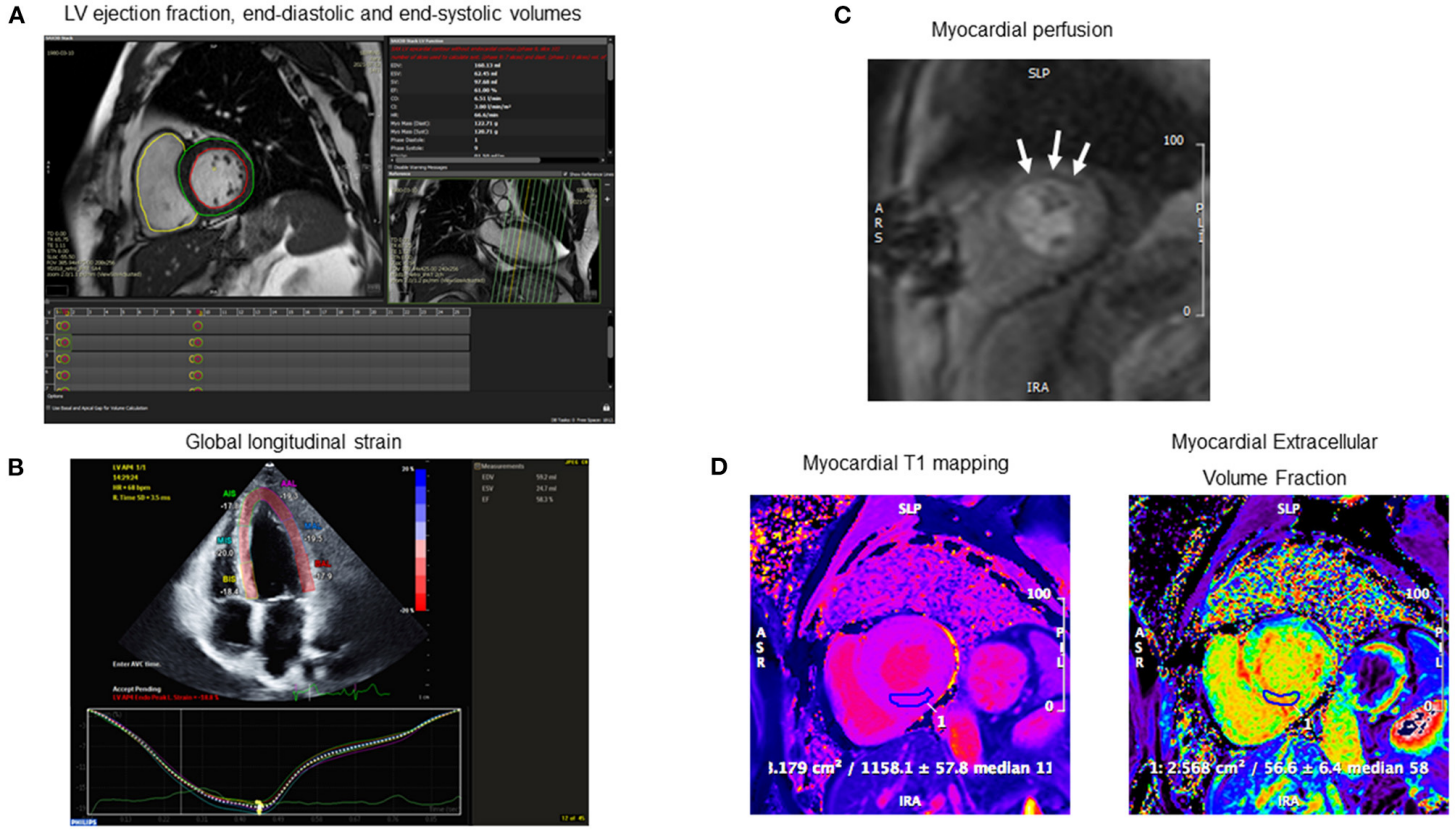
Examples of cardiac imaging metrics to assess the effects of exercise training in patients with or at risk for cardiovascular disease. **(A)** Left ventricular volumetric analysis of short-axis steady state free precession (SSFP) images acquired from cardiac MRI. **(B)** Global longitudinal strain acquired transthoracic echocardiogram. **(C)** Myocardial perfusion acquired from first-pass gadolinium enhanced imaging on vasodilator stress cardiac MRI. Note anterolateral perfusion defect (white arrows). **(D)** Myocardial tissue characterization on cardiac MRI with native T1 mapping (left) and extracellular volume fraction (ECV) (right). Note the elevated myocardial T1 (1,158 ms) and ECV (57%) consistent with cardiac amyloidosis.

Imaging provides a valuable modality to quantify the effects of exercise training and multimodal rehabilitation as it enables an evaluation of the physiological and morphological adaptations of the heart and vasculature. Imaging is frequently used to characterize the impact of exercise training interventions on cardiac function and remodeling, two prognostic measures that help to guide the therapeutic management of patients with cardiac disease. Echocardiography and magnetic resonance imaging (MRI) are the primary imaging modalities used to evaluate the cardiovascular effects of exercise based on their accuracy, versatility, and safety profile for repeat testing. In assessing the heart, these modalities are principally used to provide information on ventricular size, mass, and function as well as hemodynamic and flow quantification. Cardiac MRI also allows the evaluation of myocardial tissue characterization, including macro- and microscopic fibrosis using quantitative mapping sequences and contrast enhanced imaging (Figure 1). In assessing the vasculature, these modalities are primarily used to evaluate vascular function, stiffness, and structure. These imaging modalities are increasingly applied in real time to evaluate dynamic changes in cardiovascular function during exercise to unmask occult dysfunction that may not be identified at rest (11, 12). Furthermore, our group has also used MRI to evaluate relevant extra-cardiac sequelae of cardiac disease such as lung water content in patients with potential heart failure, regional adipose tissue deposition as a metric of cardiovascular risk, and skeletal muscle volume and function impairments (13–15).

In this review article, we will review the potential benefits of exercise on left ventricular (LV) function and remodeling, vascular function and structure, and body composition in adult patients with or at risk for cardiovascular disease and discuss the application and utility of imaging to characterize these changes.

### Exercise Training Effects on Cardiac Structure and Function in Healthy Individuals

Before examining the role of exercise training in patients with cardiovascular disease, it is important to understand its effects in healthy individuals. Public health recommendations for the general population are to include 150 min per week of moderate-intensity aerobic exercise or 75 min if the intensity is classified as vigorous (16). Sustained (e.g., several hours) endurance exercise has been linked to increased circulating biomarkers of myocardial stress including cardiac troponin T and N-terminal-pro-B-type natriuretic peptide (NT-pro-BNP) (17). However, it has been postulated that BNP and NT-pro-BNP elevation in this setting may have cytoprotective and growth regulatory effects and thus be indicative of physiological stress rather myocardial damage (17).

Soluble suppression of tumorigenicity 2 (sST2) is an emerging prognostic biomarker that has been associated with myocardial fibrosis, inflammation, and function and is an independent predictor of long-term mortality (18). High-intensity and duration exercise such as a marathon race has been linked to increased levels of sST2 (19). This association might explain the increased incidence of myocardial scar on cardiac MRI late gadolinium enhancement imaging among athletes who practice intense endurance training compared to the general population (20). Although, the clinical significance of this finding in athletes is unclear and further studies are needed. Cardiac imaging, in particular echocardiography and MRI, have a vital role in examining geometric changes that may occur in athlete's hearts. Aerobic exercise training has been associated with LV dilation and hypertrophy, right ventricular and/or biatrial dilatation (21, 22). Therefore, to differentiate between exercise-induced physiological cardiac remodeling and cardiomyopathy, a recent meta-analysis of 27 studies defined normal reference values of biventricular size and function estimated by cardiac MRI in competitive athletes. Compared to the general population, competitive athletes generally have higher resting biventricular end-diastolic and end-systolic volumes, and stroke volume (22, 23). However, an important caveat is that most patients with cardiovascular disease will not complete the volume of aerobic exercise necessary to induce physiological remodeling.

### Influence of Sex and Age on the Effects of Exercise Training on Cardiac Function

#### Sex

Ventricular volumes and mass are lower in females compared to males however cardiac function is comparable between sexes (23). Compared to men, women of comparable training status and age, typically have 5–15% lower peak VO2 after adjustment for body weight and lean body mass (24). A primary determinant of lower peak VO2 among women is lower peak exercise cardiac output relative to men (25). Given that women demonstrate a similar heart rate during exercise as men (26, 27), lower stroke volume is the primary contributor to the attenuated cardiac output response during exercise (28, 29). Additionally, sex differences exist for myocardial remodeling after injury and cardiovascular overload (e.g., training responses) (30). A recent meta-analysis of 26 studies (*n* = 468 healthy individuals) found similar LV hypertrophy adaptations to endurance training among men and women, but that LV end-diastolic, end-systolic and stroke volumes increased more in men (31). In addition, women appear to experience greater improvements in arteriovenous oxygen difference with endurance training compared to men (32–34). The sex differences literature on exercise training responses is primarily limited to healthy adults, as the studies of patients with heart failure have been predominantly performed in men (35). Several research gaps also exist regarding potential sex differences in the vascular adaptations to endurance training (36).

Estrogen levels have been shown to play an important role in attenuation of pathologic pressure-overload LV hypertrophy but may not affect exercise-related physiological hypertrophy in rodents (37, 38). Therefore, while menopausal status may be a determinant of pathological hypertrophy between the sexes, other factors may explain differences in cardiac adaptations to exercise training (30, 39). In younger, premenopausal women, the adaptations to endurance training are more likely related to peripheral determinants as opposed to LV adaptations, as seen in men (27, 36, 40, 41). Older, post-menopausal women experience similar peripheral adaptations to endurance training but appear to have a smaller increase in peak VO2 and blood volume compared to premenopausal women (32). Spina et al. demonstrated that among older (60–70 years) women, 9–12 months of endurance training increased peak VO_2_ and arteriovenous oxygen difference, but LV end-diastolic and stroke volumes did not change (40). Nio et al. compared training responses to 12 weeks of endurance training among pre- vs. post-menopausal women and found that post-menopausal women experienced a smaller increase in peak VO_2_ and blood volume, but no differences in cardiac output, heart rate, or LV volumes compared to premenopausal women (32).

#### Age

Ventricular volumes decrease with age but systolic function is unchanged with aging (23). Cardiovascular function among older adults tends to be impaired relative to younger adults owing to a number of age-related changes including worsening cardiac mechanics, decreased responsiveness to ß-adrenergic stimulation and increased vascular and aortic stiffness (36, 42–45). Among healthy, sedentary adults, there is a rate of decline in peak VO_2_ of an average of 5–10% per decade due to both structural and functional changes in central and peripheral determinants (27, 36, 40, 46). However, endurance training can attenuate the rate of age-related decline in peak VO_2_. While endurance training increases peak VO_2_ in older men and women, the magnitude of adaptation is less in older women relative younger controls (32, 47). The attenuated training adaptations appear to be related to lower improvements to cardiac output, stroke volume, and LV function among older vs. younger adults (27, 46). Older women experience similar improvements to peak VO_2_ in response to endurance training as older men, but there appears to be a sex difference in the responsible mechanisms. Prospective studies have demonstrated that older men are more likely to experience central adaptations such as improvements in cardiac output, stroke volume, and LV systolic function after 9–12 months of endurance training (48, 49). Conversely, older women are more likely to experience peripheral adaptations such as increased arteriovenous oxygen difference with minimal concurrent improvements in stroke volume (32, 33, 40, 49). Regular aerobic exercise appears to attenuate the age-related aortic stiffening among healthy men and women (36, 46) (Table 1).

**Table 1:** Characteristics of the imaging studies reporting on the cardiac effects of exercise training.

**References**	**Study design** **(sample size)**	**Study population** **(age, %** **male)**	**Imaging type**	**Aerobic exercise prescription**	**Resistance exercise prescription**	**Exercise program duration**	**Relevant results**
**Healthy adults**							
Diaz-Canestro and Montero (31)	Meta-analysis of RCTs and non-RCTs (*n* = 468)	Healthy adults (age range 22–72, 61% male)	Predominantly echocardiography or MRI	F: 3–6 days/week I: 50–85% HRR/60–95% HR_max_/60–100% VO_2max_ T: 20–180 min	None	3–12 months	Relative to men: ↓ LVEDV, LVESV, & SV ↔ LV mass
Nio et al. (32)	Single-arm prospective study (*n* = 25)	Healthy women (age range 45–58) 11 pre-menopausal & 14 post-menopausal	Echocardiography	F: 3 days/week I: HIIT: 90–95% HR_max_ × 4 intervals of 4 + 3 min recovery	None	12 weeks	Relative to post-menopausal: ↑ VO_2peak_ ↔ LVEF & LV volume
Stratton et al. (46)	Single-arm prospective study	Healthy men 11 young (age range 24–32) 13 older (age range 60–82)	Radionuclide ventriculography	F: 4–5 days/week I: increasing to 80–85% HR_max_ by 4th month T: 45 min	None	6 months	Relative to older men: ↔ VO_2peak_, LVEDV & LVEF
Spina et al. (34)	Single-arm prospective study	15 healthy men (mean age 63) 16 healthy women (mean age 64)	Acetylene rebreather and Echocardiography	F: 5 days/week I: 60–70% to 75–85% HR_max_ T: 45 min	None	9–12 months	Relative to men: ↔ VO_2peak_ ↓ exercise SV ↑ exercise arteriovenous O2 difference
**Coronary artery disease**							
Haykowsky et al. (50)	Meta-regression of RCTs (*n* = 647)	Post-MI (mean age 55, “pre-dominantly” male)	Echocardiography, MRI or radionuclide ventriculography	F: 3–7 days/week I: ~60–85% VO_2peak_/80% HR_max_ T: 30–180 min	NR	1-6 weeks	Relative to control: ↑ LVEF ↓ LVEDV & LVESV with earlier initiation post-MI and increased program duration
Zhang et al. (51)	Meta-analysis of RCTs (*n* = 1,137)	Post-MI (mean age 58, 93% male)	Echocardiography, MRI or radionuclide ventriculography	F: 3–5 days/week I: 60–85% HRR/70–90% HR_peak_/55–85% VO_2peak_ T: 20–60 min	None	NR	Relative to control: ↑ VO_2peak_ & LVEF ↓ LVD when initiated <29 days post-MI
McGregor et al. (52)	Longitudinal, controlled trial (*n* = 56)	Post-MI with preserved LVEF (mean age 56 ± 10, 100% male)	Echocardiography	F: 2 days/week I: 60–80% VO_2peak_ T: 40 min	F: 2 days/week I: based on RPE T: 1 set × 12 reps; 25–40 min	10 weeks	Relative to control: ↑ VO_2peak_ ↓ LVEDV & LVESV
Gaillauria et al. (53)	RCT (*n* = 61)	Post-MI with reduced LVEF (mean age 55 ± 3, 72% male)	Echocardiography	F: 3 days/week I: 60-70% VO_2peak_ T: 40 min	NR	6 months	Relative to control: ↑ VO_2peak_ & LVEF ↓ LVEDV & LVESV
Gaillauria et al. (54)	RCT (*n* = 46)	Post-acute ST elevation MI (mean age 54 ± 8, 87% male)	Echocardiography	F: 3 days/week I: 60–70% VO_2peak_ T: 30 min	NR	6 months	Relative to control: ↑ VO_2peak_ ↑ LVEF
Haddadzadeh et al. (55)	RCT (*n* = 42)	Post-coronary event (mean age 62 ± 9, 77% male)	Echocardiography	F: 3–5 days/week Center-based group I: 40–70% HRR T: 20–40 min Home-based group I: 40–70% HRR T: 20–40 min	None	12 weeks	Center and home-based groups relative to control: ↑ LVEF Center-based group relative to home-based group: ↔ LVEF
Belardinelli et al. (56)	RCT (*n* = 46)	CAD & reduced LVEF (mean age 57 ± 9, 91% male)	Dobutamine stress echocardiography followed by thallium myocardial scintigraphy	F: 3 days/week I: 60% VO_2peak_ T: 40 min	None	8 weeks	Relative to control: ↑ VO_2peak_ & contractile response to dobutamine and thallium activity
Belardinelli et al. (57)	RCT (*n* = 30)	CAD & reduced LVEF (mean age 55 ± 9, 100% male)	Dobutamine stress echocardiography followed by thallium myocardial scintigraphy	Dipyridamole (75 mg/day) given with exercise F: 3 days/week I: 60% VO_2peak_ T: 30 min	None	8 weeks	Relative to control: ↑ VO_2peak_, coronary collateral score, thallium activity, LVEF, & WTSI
Gaillauria et al. (58)	RCT (*n* = 50)	Post-acute ST elevation MI (mean age 53 ± 9, 92% male)	Gated single-photon emission computed tomography imaging	F: 3 days/week I: 60–70% VO_2peak_ T: 40 min	None	6 months	Relative to control: ↑ VO_2peak_ ↓ resting & stress WMSI, resting & stress WTSI
**Heart failure**							
Chen et al. (59)	Meta-analysis of RCTs (*n* = 813)	Heart failure with reduced EF (age range 54–74, “pre-dominantly male”)	Echocardiography or MRI	F: 2–5 days/week I: 60–80% VO_2peak_ T: 20–60 min	Too few studies for comparison	Most 3–6 months, range 2–14 months	Relative to control: ↑ LVEF ↓ LVEDV & LVESV with greater effect for ≥ 6 months training
Erbs et al. (60)	Retrospective analysis of RCT (*n* = 73)	Chronic heart failure as a result of dilative cardiomyopathy or ischemic heart disease (mean age 53 ± 3)	Echocardiography	F: 7 days/week I: 70% VO_2peak_ T: 20 min	F: 1 time/week I: Group training (walking, calisthenics, & non-competitive ball games) T: 60 min	6 months	Relative to control: ↑ VO_2peak_ ↓ LVEDD
Erbs et al. (61)	RCT (*n* = 37)	Chronic heart failure as a result of dilative cardiomyopathy or ischemic heart disease (mean age 61 ± 11, 100% male)	Echocardiography	F: 7 days/week I: 60% VO_2peak_ T: 20–30 min	F: 1 days/week I: Group training (walking, calisthenics, & non-competitive ball games) T: 60 min	12 weeks	Relative to control: ↑ VO_2max_ & LVEF ↓ LVEDV, LVESV, LVEDD, & LVESD
Tucker et al. (62)	Meta-analysis of RCTs (*n* = 1,078)	Heart failure with reduced EF (mean age 63, 77% male)	Echocardiography or MRI	F: 3–5 days/week HIIT group: I & T: 85–95% HR_peak_/VO_2peak_ 3–6 min intervals + 2–3 min recovery (25–47 min total) MIT group: I & T: 40–70% HRR/77–90% HR_max_/60–80% VO_2peak_ for 20–45 min	Too few studies for comparison	Most 3-6 months, range 1 month to 10 years	Relative to control: ↑ LVEF & VO_2peak_ with greater effect for ≥ 6 months training No difference in change in LVEF between HIIT & MIT groups ↔ LVEDV & LVESV
Pearson et al. (63)	Meta-analysis of RCTs and non-RCTs (*n* = 470)	Heart failure (any type) (age range 49–77, “pre-dominantly male”)	Echocardiography	F: 2–7 days/week I: 40–80% HRR/45–70%HR_max_/60–80% VO_2peak_ T: 20–60 min	F: 2–3 days/week I: 50–80% 1RM T: 2–3 sets × 8–10 reps	1–7 months	Relative to control: ↓ LV E/e'
Hambrecht et al. (64)	RCT (*n* = 73)	Heart failure with reduced EF (mean age 54 ± 9)	Echocardiography	F: 7 days/week I: 70% VO_2peak_ T: 20 min	F: 1 time/week I: Group training (walking, calisthenics, and non-competitive ball games) T: 60 min	6 months	Relative to control: ↑ LVEF ↓ LVEDD
Kitzman et al. (65)	RCT (*n* = 63)	Heart failure with preserved EF (mean age 70 ± 7, 24% male)	Echocardiography	F: 3 days/week I: 40–70% HRR T: 60 min	None	16 weeks	Relative to control: ↑ VO_2peak_ ↔ LVEDV, LVESV, LVEF, & LV E/A ratio
Haykowsky et al. (35)	RCT (*n* = 40)	Heart failure with preserved EF (mean age 69 ± 6, 12% male)	Echocardiography	F: 3 days/week I: 40–70% HRR T: 60 min	None	16 weeks	Relative to control: ↑ VO_2peak_ ↔ LVEDV
Mueller et al. (66)	RCT (*n* = 176)	Heart failure with preserved EF (age 70 ± 8, 33% males)	Echocardiography	HIIT group F: 3 days/week I: 4 × 4-min intervals @ 80–90% HRR T: 38 min MIT group F: 5 days/week I: 35–50% HRR T: 40 min	None	12 weeks	HIIT & MIT relative to control: ↑ VO_2peak_ ↔ LV E/e'
Fukuta et al. (67)	Meta-analysis of RCTs (*n* = 436)	Heart failure with preserved EF (mean age 66, 37% male)	Echocardiography or Doppler ultrasound	F: 2–3 days/week I: 70% HRR/60–75% HR_max_/60–80% VO_2peak_ T: 20–60 min	None	3–6 months	Relative to control: ↑ VO_2peak_, ↔ LVEF, E wave, & E/e'
**Patients at risk for cardiovascular disease**							
Leggio et al. (68)	Single-arm prospective study (*n* = 116)	Hypertensive (mean age 51 ± 8, 49% male)	Echocardiography	F: 3 days/week I: 70–85% VO_2peak_ T: 45 min	None	8 weeks	Relative to baseline: ↑ LV S' ↓ LV E/e' ↔ LVEF, LV mass
Molmen-Hansen et al. (69)	RCT (*n* = 88)	Hypertensive (mean age 52 ± 8, 56% male)	Echocardiography	F: 3 days/week HIIT group: I & T: 4 × 4-min @85–90% VO_2max_ T: 38 min MIT group: I: 70% VO_2max_ T: 47 min	None	12 weeks	HIIT and MIT relative to control: ↑ VO_2*max*_ HIIT relative to MIT: ↑ LVEF, LV e' & S' ↔ LV E & A wave, LV wall thickness
Sahin et al. (70)	RCT (*n* = 30)	Hypertensive (mean age 56 ± 9, 38% male)	Echocardiography	F: 3 days/week I: alternating 1-min low & high load T: 20 min	F: 3 days/week I: 75% 1-RM T: 6 exercises (3 upper/3 lower extremities), 3 sets × 10 reps	12 weeks	Relative to control: ↑ LV GLS & LA reservoir strain ↓ LV mass
Verboven et al. (71)	Systematic Review of RCTs and prospective studies (*n* = 705)	Type 2 diabetes (mean age range 46–61, sex not reported)	Echocardiography or MRI	F: 2–4 days/week I: HIIT: 90–95% HR_max_ MIT: 50–75% HR_max_	F: 2–3 days/week I: 55–80% of maximum voluntary contraction (*n* = 59)	12 weeks−1 year	Relative to MIT: ↑ VO_2peak_, LV E/A ↔ LV GLS, LVEF, LV mass
Cassidy et al. (72)	RCT (*n* = 28)	Type 2 diabetes (mean age 60 ± 9, 78% male)	MRI	F: 3 days/week I&T: 5 × 2–4-min @RPE 16–17 with 3-min recovery T: 33 min	None	12 weeks	Relative to control: ↑ LV mass, LVEDV, LVSV, LVEF, & LV E wave
Hollekim-Strand et al. (73)	Pilot RCT (*n* = 47)	Type 2 diabetes with LV diastolic dysfunction (mean age 56 ± 6, 64% male)	Echocardiography	HIIT group: F: 3 days/week I & T: 4 × 4-min @90–95% HR_max_ for 40 min MIT group: F & I: NR T: >10 min bouts, 210 min/week	None	12 weeks	HIIT relative to MIT: ↑ VO_2peak_, LV e', LV S' ↔ LV E/e'
Murray et al. (74)	Systematic review of RCTs and non-RCTs (*n* = 221)	Breast cancer (during chemotherapy) (mean age 49, 0% males)	Echocardiography	F: 3 days/week I: 70% HRR/50–95% HR_max_/60–100% VO_2peak_ T: 15–60 min	F: 3 days/week I: NR T: 60 min in combination with aerobic training	1–16 weeks	Relative to control: ↑ VO_2peak_ ↔ LV GLS & LVEF

*A, peak mitral inflow during atrial systole; E, early diastolic transmitral velocity; e', early diastolic mitral annular velocity; EDD, end-diastolic diameter; EDV, end-diastolic volume; EF, ejection fraction; ESD, end-systolic diameter; ESV, end-systolic volume; F, frequency; GLS, global longitudinal strain; HIIT, high intensity interval training; HR_max_, max heart rate; HRR, heart rate reserve; I= intensity; LA: left atrial; LV, left ventricular; MIT, moderate intensity training; MRI, magnetic resonance imaging; MVC, maximal voluntary contraction; RCTs, randomized controlled trials; RPE, rate of perceived exertion; T, time/duration; S', systolic mitral annulus tissue velocity; SBP, systolic blood pressure; VO_2peak_, peak volume of oxygen consumption with exercise; VO_2max_, max volume of oxygen consumption with exercise; WMSI, wall motion score index; WTSI, wall thickening score index; 1-RM, 1-rep max*.

## Exercise Training Effects on Cardiac Structure and Function in Patients With Established Cardiovascular Disease

### Cardiac Remodeling in Cardiovascular Disease States: Clinical Significance

Left ventricular remodeling after myocardial injury is defined as a structural adaptation in chamber size and shape, arising from complex biochemical and cellular changes. This deleterious cascade leads to varying degrees of LV dilatation, hypertrophy and extra-cellular collagen deposition, manifesting clinically as myocardial stiffness, and/or LV dysfunction (75). The extent of remodeling is often used as a surrogate for cardiac disease progression and is an emerging therapeutic target in heart failure. Therapeutic interventions, including cardiac rehabilitation, have been used to attenuate cardiac remodeling, typically defined as a ≥10% increase in LV end-diastolic or end-systolic volume in the post-myocardial infarction (MI) or heart failure setting (76). Cardiac imaging routinely provides data on remodeling-specific markers, such as LV volumes and ejection fraction (EF), which elucidate prognosis and guide the potential need for further intervention (76).

Studies of structured, exercise-based cardiac rehabilitation for patients with heart failure or post-MI have been associated with improved cardiac function and attenuation of ventricular remodeling in addition to a reduction in cardiovascular mortality (77, 78). Thus, these programs have been implemented as a class I recommendation in contemporary guidelines for patients with cardiac diseases (1–4). Cardiac imaging is commonly used to evaluate the effects of exercise training on LV size and function in conjunction with other therapeutic interventions such as pharmacotherapy, coronary revascularization and cardiac resynchronization.

### Exercise Training Effects in Patients With Coronary Artery Disease

Echocardiography has been the predominant modality used to study the effects of exercise training on cardiac remodeling in patients with CAD. Pooled analyses of clinical trial data suggest that the timing and duration of the exercise intervention is important. For example, these studies have shown that the attenuation of cardiac remodeling occurs when exercise is initiated early, within 1 week of hospitalization in clinically stable, post-MI patients and is continued for at least 6 months (50, 51). These salient effects on LV remodeling post-MI have been described in patients with preserved LV function (52) as well as moderate systolic dysfunction (53). Randomized controlled trials of exercise training have demonstrated improvements in LVEF ranging from 5 to 15% (51, 54, 55). There is also preliminary evidence to suggest that exercise may improve diastolic function. A randomized controlled trial evaluating the effects of exercise on diastolic function post MI found that E wave and E/A ratio by Doppler echocardiography increased by 0.2 in the exercise group, suggesting enhanced LV filling and reduced LV wall stress (53).

Exercise training and cardiac rehabilitation are effective strategies for limiting morbidity and mortality inpatients with coronary artery disease (77, 78). The mechanisms underlying this reduction in mortality are likely multifactorial but include reductions in cardiovascular risk factors as well as improved myocardial perfusion, which can be attributed to increased coronary artery collateralization and vasorelaxation as well as reduced vascular oxidative stress (79, 80). Improved coronary artery collateralization secondary to exercise training has been demonstrate using Thallium scintigraphy and gated SPECT with technetium-99 m sestamibi (56–58, 81). Coronary autoregulation is influenced by the interplay between nitric oxide production by the endothelium and inactivation by reactive oxygen species. In patients with CAD, coronary vasomotor tone is disturbed due to an imbalance in nitric oxide metabolism, however, exercise training restores nitric oxide availability and is largely responsible for the improvement in myocardial perfusion (81).

In summary, imaging studies demonstrate that exercise training has favorable effects on LV remodeling, systolic and diastolic function as well as myocardial perfusion in patients with CAD (Table 1).

### Exercise Training Effects in Patients With Heart Failure

In patients with heart failure and reduced ejection fraction (HFrEF) (LVEF < 40%), exercise training has been shown to reverse LV remodeling, with favorable effects on ejection fraction, stroke volume, and end-diastolic and end-systolic diameters (59–62).

However, similar to patients with CAD, the length of training program needed to have measurable positive effects was 6 months or longer (59, 60, 62). In one recent meta-analysis, LVEF was observed to improve with a mean difference of 6.3% in trials of ≥ 6 months duration vs. 2.3% for studies of shorter duration (62). Similarly, clinical trials of patients with reduced ejection fraction (LVEF < 35%) found that exercise training of 3 months duration or less did not impact relevant circulating cardiac biomarkers: NT-pro-BNP, high sensitivity C-reactive protein, and cardiac troponin (82). Regarding the type of exercise, the meta-analysis reported that high intensity interval training (HIIT) or combined aerobic and resistance training was not superior to moderate intensity continuous training for improvement in LVEF (62). Diastolic function also likely improves with exercise training. A meta-analysis of patients with HFrEF found that aerobic training induced a mean difference of −2.85 in E/E' ratio (early diastolic filling over diastolic tissue velocity) relative to controls, suggesting improved myocardial relaxation (63). However, this exercise-induced improvement in cardiac function is possibly explained by changes to vascular tone rather than direct cardiac effects. Using echocardiography and right-heart catheterization, Hambrecht et al. showed that 6 months of training in patients with HFrEF lowered total peripheral resistance, which strongly correlated to concurrent increased stroke volume (64). In studies of patients with heart failure and preserved ejection fraction (HFpEF) (LVEF ≥ 50%), exercise training did not result in any significant changes in LV volume, systolic function and diastolic function but did lead to a mild improvement in cardiorespiratory fitness [improves peak oxygen uptake (VO_2_) by ~2 ml/kg/min] (65). Furthermore, measurements of carotid artery distensibility and brachial artery flow mediated dilatation were also unaffected by aerobic training in this study (65). However, exercise trained patients with HFpEF exhibit increased arterial-venous oxygen difference after 4 months, consistent with improved skeletal muscle oxygen uptake and/or extraction by active muscles (35). A recent randomized controlled trial of HIIT, moderate intensity intermittent training or guideline-based control in 180 patients with HFpEF confirmed no improvement in echo-derived diastolic function (E/E') and left atrial volume or cardiorespiratory fitness (peak VO_2_) after 12 months of training (66). An earlier meta-analysis also reported no change in cardiac imaging parameters and minor improvement in exercise performance in patients with HFpEF (67) (Table 1).

## Exercise Training Effects on Cardiac Structure and Function in Patients at Risk for Cardiovascular Disease

### Patients With Hypertension

Uncontrolled systemic hypertension may lead to LV hypertrophy and ultimately to heart failure (83). Moderate-intensity aerobic exercise training is recommended in patients with hypertension has been associated with 2-3 mmHg reductions in both systolic and diastolic blood pressure (84). Single-site imaging studies of exercise training in hypertensive patients also suggest associated improvement in cardiac function. An observational study of 116 patients undergoing 8 weeks of moderate-to-high intensity aerobic training (70–85% of peak heart rate) found no change in LVEF or LV wall thickness however tissue Doppler echocardiography and strain imaging suggested a post-intervention improvement in systolic and diastolic function (68). In a randomized study of 88 patients with hypertension, Molmen-Hansen et al. showed that high-intensity interval training (>90% maximal heart rate) improved 24-h ambulatory blood pressure, increased LVEF from 58 to 65%, increased E' from 8.06 to 9.26 cm/s, but with no change in LV wall thickness (69). A recent study of 30 hypertensive patients participating in 10 weeks of cardiac rehabilitation improved blood pressure control, decreased LV mass from 104 to 97 g/m^2^ and improved LV global longitudinal strain from 19.8 to 20.7% (70). Interestingly, a meta-analysis of 16 studies of normotensive and hypertensive patients showed that isometric resistance training lowered systolic blood pressure in both groups, however potential associated effects on cardiac structure and function have not been elucidated (85) (Table 1).

### Patients With Diabetes Mellitus

Diabetes mellitus is an independent risk factor for the development of atherosclerosis and heart failure. Diabetic cardiomyopathy is characterized by two distinct phenotypes, LV dilatation with systolic dysfunction or concentric LV hypertrophy with diastolic dysfunction (86). Glycemic control has been shown to influence cardiac function in individuals with diabetes (87), however, even individuals with well-controlled diabetes (defined by HbA1c < 7.5%) have subclinical myocardial dysfunction demonstrated by a significantly reduced longitudinal strain reserve on stress echocardiography (88). A systematic review of exercise training in individuals with diabetes suggested an improvement in diastolic function (E/A) but no consistent effect on systolic function or LV remodeling on echocardiography or MRI (71). Nevertheless, this apparent effect of exercise training is likely important because diastolic dysfunction with preserved LVEF is a common phenotype in patients with diabetic cardiomyopathy (86).

Two studies have suggested that high-intensity intermittent exercise may be superior to moderate-intensity exercise in reversing diabetes-associated myocardial impairment, with demonstrated improvement in systolic and diastolic function (72, 73). However, high-intensity training also increases LV wall mass and end-diastolic volume, presumably due to changes in loading conditions (72) (Table 1).

### Patients With Cancer

Cancer therapy-related cardiac dysfunction (CTRCD) is an important clinical consideration for patients receiving anthracycline and/or trastuzumab-based adjuvant treatment. Early recognition and management of CTRCD is important as it has the potential to delay cancer treatment and directly impact cardiovascular morbidity and mortality. Cardiac imaging plays a critical role in the early detection and monitoring of CTRCD. This adverse complication is most commonly assessed by measuring LVEF by echocardiography, multi-gated blood pool imaging or cardiac MRI (89). Left ventricular global longitudinal strain is also recommended as a sensitive metric to. detect early LV dysfunction (89).

While pre-clinical models suggest that aerobic exercise training prevents cancer therapy-related cardiotoxicity, this has not translated to the clinical setting in the available research to-date. A recent meta-analysis of 8 studies (221 patients) of exercise training in patients with breast cancer receiving trastuzumab and/or anthracyclines showed no consistent benefit in the prevention of LV dilatation or worsening systolic function as assessed by echocardiography or cardiac MRI (74). However, exercise did improve cardio-respiratory fitness as measured by peak VO_2_ (Table 1).

## Cardiac Imaging With Exercise Stress

Cardiac reserve function is defined as the difference in cardiac function from rest to peak exercise captured by echocardiography or cardiac MRI. Its potential importance stems from its ability to detect stress-induced myocardial dysfunction and evaluate exercise-related changes to chronotropy, inotropy, lusitropy, and vasodilation in patients with normal resting parameters (90).

In patients with HFpEF, a number of metrics have been studied with exercise imaging including LVEF, LV global longitudinal strain, E/E' ratio, mitral annular systolic velocity, and tricuspid regurgitation maximal velocity (91–93). Resting echocardiography, electrocardiogram, and serum biomarkers such as NT-pro-BNP often fail to identify patients with HFpEF, whereas a stress echocardiogram has been shown to be more sensitive for this diagnosis (93). Exercise echocardiography can be performed using a treadmill or a cycle ergometer. The latter having the advantage of imaging being performed in a semi-supine position and therefore does not require patient transfer and allows for image acquisition at incremental workloads.

**Figure 2:**
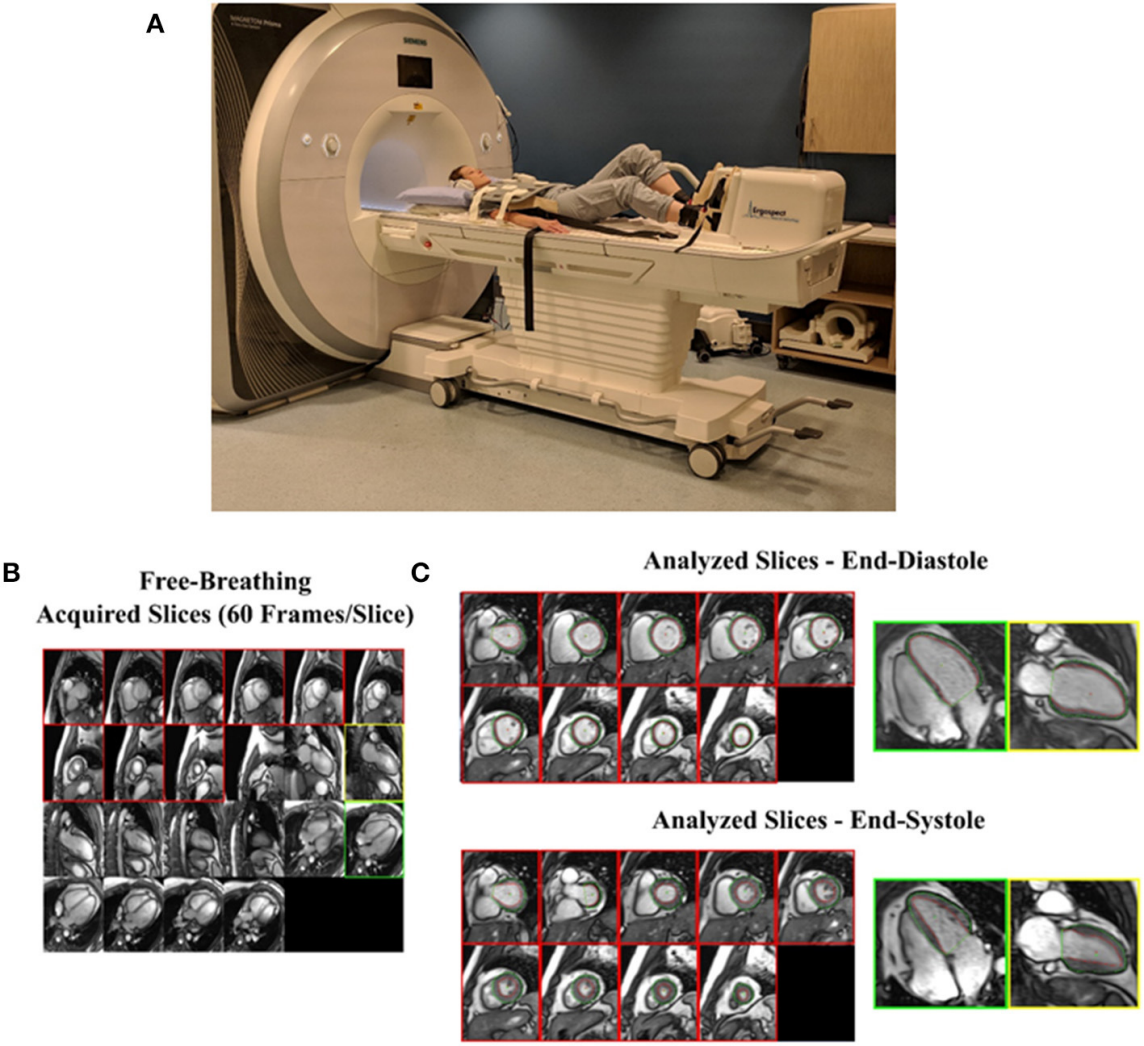
Real-time imaging of cardiac function during exercise using cardiac MRI. **(A)** Healthy volunteer in supine position outside of magnet bore using MRI conditional stepper device to achieve maximal aerobic activity. **(B)** All short-axis and long-axis slices are viewed simultaneously to select those for volumetric analysis. Short-axis slices with myocardium and a single 2- and 4-chamber view are chosen. **(C)** A full cardiac cycle for each selected slice is extracted from which end-diastolic and end-systolic images are identified and endocardial (red) and epicardial (green) borders are traced. Modified from Kirkham et al. (97).

Exercise stress imaging with cardiac MRI offers the advance of enhanced accuracy and reproducible quantification of cardiac volumes and biventricular function relative to other imaging modalities (94, 95). The exercise modes employed with cardiac MRI include a supine cycle ergometer or stepper device attached to the scan table to allowing real-time image acquisition while the patient is exercising, instantaneous with achievement of peak exercise or by exercise performed outside of the bore with quick transfer of patients to the MRI table for image acquisition (96, 97). Exercise-related increased respiratory rates and cardiac translation introduce both patient-related difficulties in breath-holds and ECG signal, which are required for the standard cardiac MRI acquisition methods (97). Our group recently validated a real-time, free-breathing approach that remedies these exercise-related issues in patients with cardiovascular risk factors and/or cancer (97) (Figure 2).

## Exercise Training Effects on Vascular Health

### Imaging Metrics of Vascular Structure and Function

**Figure 3:**
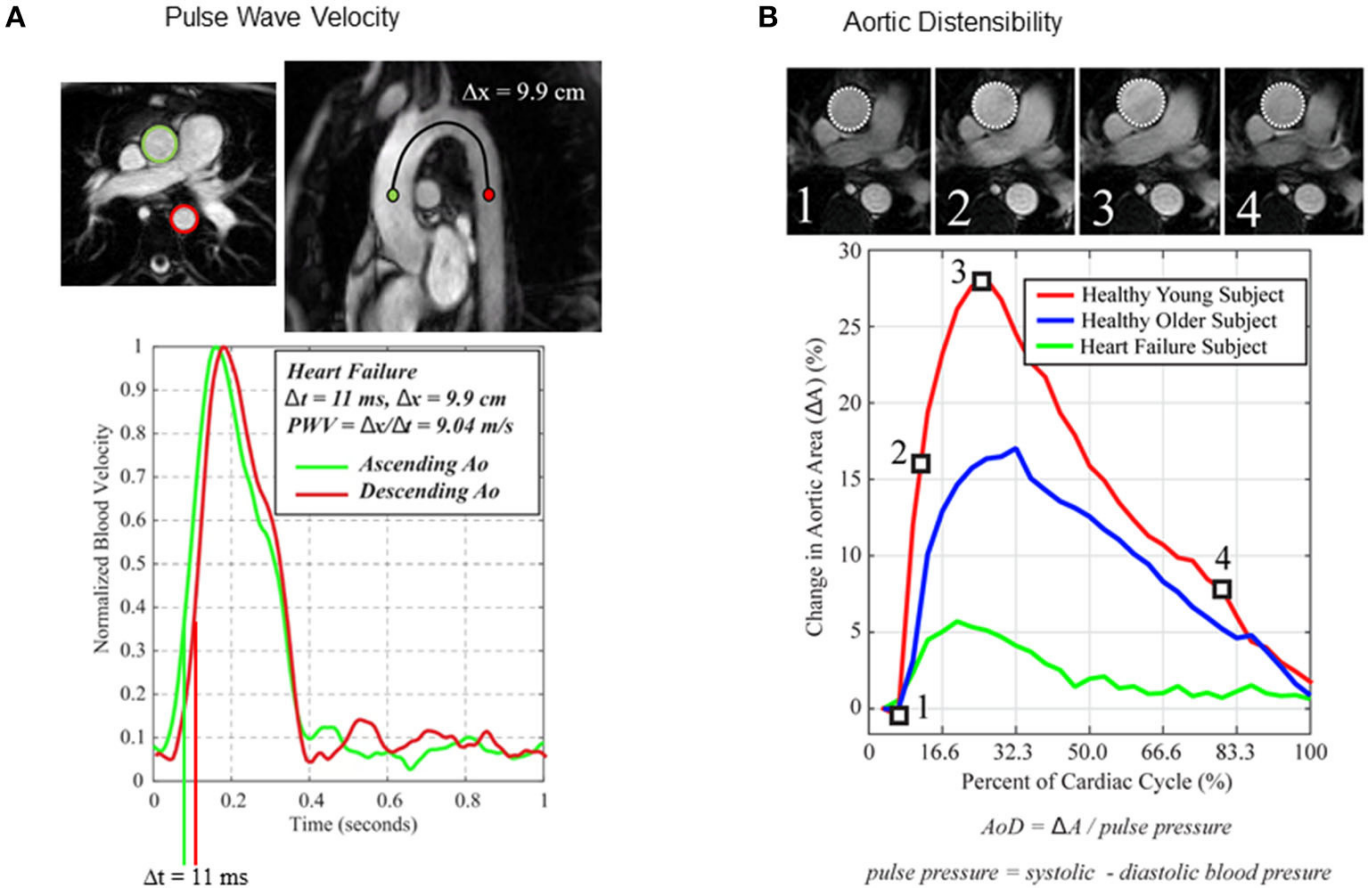
Imaging techniques used to assess the effects of exercise training in patients with or at risk for cardiovascular disease. **(A)** MRI derived arterial stiffness. Pulse wave velocity (PWV) estimated from MRI derived phase velocity imaging of the thoracic aorta in an older patient with heart failure. Adapted from Thompson et al. (101). **(B)** Thoracic aorta distensibility (AoD) on SSFP cines. Comparison of aortic distensibility between a young healthy individual, an older healthy individual and an older patient with heart failure.

Age, cardiometabolic disease and toxic exposures (e.g., smoking, chemotherapy) each affect the arterial wall matrix, reduce elasticity and thus increase the potential for arterial stiffening and impaired blood flow (98). Endothelial dysfunction is also common in patients with and at risk for atherosclerotic disease and can increase arterial tone and stiffness due to impaired vascular smooth muscle function (98). Arterial stiffness is typically derived from the assessment of pulse wave velocity (PWV) in a vessel segment and is measured non-invasively using tonometry or cuff-based technologies as well as by imaging on Doppler ultrasound or MRI-derived phase velocity imaging (99). These imaging modalities also allow for complementary information on arterial wall thickness (B-mode or M-mode ultrasound) (100) and aortic distensibility (MRI cine imaging) (101) (Figure 3). Measures of arterial stiffness are clinically relevant as they predict future cardiovascular events (102–104) incident hypertension (105) and heart failure (106). Hence, therapies directed at attenuating vascular dysfunction are desirable. Aerobic exercise training is known to improve cardiovascular risk factors modulating arterial stiffness (4) and some studies also suggest direct effects on improving vascular function (107, 108).

## Exercise Training Effects on Vascular Function in Patients With Established Cardiovascular Disease

### Patients With Coronary Artery Disease

Aerobic exercise training has been linked to reduced arterial stiffness in patients with CAD. A systematic review of 5 studies of arterial stiffness in patients with CAD found that aerobic training was associated with lower PWV measures on Doppler ultrasound (109). One study suggested that the magnitude of benefit was greater for participants who trained for 20 weeks compared to 12 weeks, change in PWV −1.0 vs. −0.6 m/s, respectively (110). However, these studies were non-randomized, small (range 20–119 participants) and did not control for potential confounders. In contrast, a randomized-controlled trial of 12 months of combined aerobic and resistance training in 137 patients with CAD and diabetes mellitus found no effect of exercise on IMT measures (111). Although, subgroup analysis suggested improved IMT measures in patients without carotid plaques at baseline (Table 2).

**Table 2:** Characteristics of the imaging studies reporting on the vascular effects of exercise training.

**References**	**Study design** **(sample size)**	**Study population** **(age, %** **male)**	**Imaging type**	**Aerobic exercise prescription**	**Resistance exercise prescription**	**Exercise program duration**	**Relevant results**
**Coronary artery disease**							
Oliveira et al. (109)	Systematic review of prospective studies (*n* = 271)	CAD (mean age range 48–67, sex not reported)	Carotid-femoral or carotid-brachial Doppler ultrasound	F: 1–3 days/week I: 40–85% HRR/anaerobic threshold T: 15–50 min	Too few studies for comparison	6–20 weeks	Relative to control: ↓ PWV
Laskey et al. (110)	Prospective, single-arm trial (*n* = 48)	Clinically stable CAD (mean age 61 ± 11, 54% male)	Doppler ultrasound	F: 3 days/week I: 40–85% HRR T: 15–50 min	None	20 weeks	Relative to control: ↓ central aortic systolic pressure & PWV
Byrkjeland et al. (111)	RCT (*n* = 137)	Type 2 diabetes and CAD (mean age 63 ± 8, 84% male)	Ultrasonography	F: 3 days/week I: not reported T: 33 min	F: 3 days/week I: not reported T: 17 min	12 months	Relative to control: ↔ carotid intima-media thickness
**Heart failure**							
Kitzman et al. (65)	RCT (*n* = 63)	Heart failure with preserved ejection fraction	Ultrasonography, Doppler echocardiography	F: 3 days/week I: 40–70% HRR T: 60 min	None	16 weeks	Relative to control: ↑ VO_2peak_ ↔ carotid artery distensibility & FMD
**Patients at risk for cardiovascular disease**							
Madden et al. (108)	RCT (*n* = 36)	Type 2 diabetes, hypertension and hypercholesterolemia (mean age 71 ± 1, 52% male)	Doppler ultrasound	F: 3×/wk I: 60–75% HRR T: 60 min	None	12 weeks	Relative to control: ↓ femoral and radial PWV ↔ VO_2*peak*_
Madden et al. (112)	RCT (*n* = 52)	Type 2 diabetes, hypertension and hypercholesterolemia (mean age 69 ± 1, 58% male)	Doppler ultrasound	F: 3×/wk I: 60–75% HRR T: 60 min	None	6 months	Relative to control: ↓ femoral and radial PWV ↑VO_2*peak*_
Montero et al. (113)	Meta-analysis of RCTs & non-RCTs (*n* = 472)	Pre-hypertensive (age range 44–70, <50% males)	Doppler ultrasound	F: 3–6/wk I: 60–75% HRR/60–85%HR_max_/50–75% VO_2max_ T: 25–60 min	None	1–7 months	Relative to control: ↔ measures of arterial stiffness (PMV, distensibility)
Way et al. (114)	Meta-analysis of RCTs & non-RCTs (*n* = 783)	Type 2 diabetes (mean age 57 ± 17, 51% male)	Doppler ultrasound	F: 3×/wk I: 60–75% HRR/60–90% HR_max_/50–85% VO_2peak_/65–75% VO_2max_ T: 25–60 min	F: 2–3×/wk I: 50–70% 1–RM/60–80% MVC T: 2–3 sets × 10–15 reps, 25–60 min	12–24 weeks	Relative to control: ↓ endothelial-independent dilation ↔ PWV & FMD
Jones et al. (115)	RCT (*n* = 51)	Breast cancer survivors (mean age 56 ± 7, 0% male)	Doppler ultrasound	F: 2×x/wk I: 50–70% HR_max_ T: 60 min	F: 2×/wk I: 60% 1-RM T: 12 resistance-based exercises, 1 set × 10–12 reps	12 weeks	Relative to control: ↓ PWV ↑VO_2*max*_
Beaudry et al. (116)	Meta-analysis of RCTs (*n* = 163)	Cancer survivors (mean age 57 ± 7, 54% male)	Ultrasonography	F: 3×/wk I: 55–75% VO_2peak_ T: 20–45 min	None	3–6 months	Relative to control: ↑ FMD & VO_2peak_

*CAD, coronary artery disease; F, frequency; FMD, flow mediated dilation; HR_max_, max heart rate; HRR, heart rate reserve; I, intensity; LV, left ventricular; MVC, maximal voluntary contraction; NMD, nitric-flow mediated dilation; PWV, pulse wave velocity; RCTs, randomized control trials; T, time/duration; VO_2peak_, peak volume of oxygen consumption with exercise; VO_2max_, max volume of oxygen consumption with exercise; 1-RM, 1-rep max*.

### Patients With Heart Failure

Vascular function is believed to play an integral role in the development and progression of heart failure, particularly with preserved ejection fraction (117). Indeed, carotid artery distensibility derived from B-mode ultrasound is significantly reduced in patients with HFpEF compared to healthy controls and is also directly related to cardiorespiratory fitness (peak VO_2_) (118). In a cross-sectional study of 143 patients attending cardiac rehabilitation, a significant correlation between indices of arterial stiffness and cardiorespiratory exercise tolerance was found in patients with preserved ejection fraction but not in patients with reduced ejection fraction (119). However, a randomized, controlled trial of 16 weeks of combined aerobic and resistance training for patients with HFpEF found no improvement in carotid artery distensibility or in brachial artery flow mediated dilatation despite improvement in cardiorespiratory fitness (65). While vascular function appears to be an important determinant of exercise tolerance in patients with HFpEF, it does not appear to be improved by exercising training (Table 2).

## Exercise Training Effects on Vascular Function in Patients at Risk for Cardiovascular Disease

Early studies suggested that 3 months of aerobic exercise training in patients with cardiovascular risk factors (hypertension, diabetes mellitus and dyslipidemia) reduced arterial PWV (108). However, these salient effects on arterial stiffness were an early response to exercise as they were not sustained at 6 months of aerobic training (112). Similarly, meta-analyses of predominantly aerobic exercise training in individuals with hypertension or diabetes found no improvement in the non-invasive assessment of arterial stiffness (113, 114). However, subgroup analysis in hypertensive patients suggested improvement with interventions of longer duration (113). Data on exercise training and arterial stiffness in patients with cancer is lacking however one recent study of 12 weeks of circuit resistance training reported significant improvement in cardiorespiratory fitness (VO_2_ max + 4.3 ml/kg/min) and PWV (−0.9 m/s) in 51 patients (115). A recent meta-analysis of exercise training in patients with breast or prostate cancer (163 total patients) reported that aerobic exercise improved vascular endothelial function on ultrasound (116) (Table 2).

## Exercise Training Effects on Cardiac and Extra-Cardiac Tissue Structure and Composition

Late gadolinium enhancement sequences on cardiac MRI are frequently utilized to identify and quantify myocardial scar (replacement fibrosis) arising from acute ischemic injury (120). Follow-up studies of patients with acute MI, have shown that infarct size decreases by 16% on late gadolinium enhancement imaging (121). However, the effect of exercise training on infarct size has not been well-elucidated. Quantitative mapping MRI sequences also allow the characterization of both myocardial and extra-cardiac tissues to provide valuable insight into whole-body cardiovascular health profile. Elevated myocardial T_1_ times and extracellular volume metrics have been validated as indicators of edema in acute disease and interstitial reactive fibrosis in chronic conditions and are increasingly used as risk markers in patients at risk for or with established cardiovascular disease (122, 123). For example, Reinstadler et al. showed that in patients with ST elevation myocardial infarction, native T_1_ values in remote, non-infarcted myocardium independently predicted adverse cardiovascular outcomes at 6 months (124). Similarly, Vita et al. showed that in patients with non-ischemic dilated cardiomyopathy, elevated myocardial extracellular volume predicts long-term heart failure outcomes (125). Increased myocardial T_1_ and extracellular volume have also been reported in cancer survivors with previous exposure to anthracycline-based chemotherapy (126, 127). Kirkham et al., reported that native myocardial T_1_ was elevated compared to controls and correlated with cardiorespiratory fitness among anthracycline-treated breast cancer survivors, suggesting that this metric of myocardial microarchitecture has important functional implications (128). A recent non-randomized study of 27 patients with breast cancer undergoing 4 months of exercise training during anthracycline-based chemotherapy found no effect of exercise on native myocardial T_1_ but not report on extracellular volume fraction (129). To our knowledge, there have been no reports on the effects of exercise training on myocardial T_1_ and extracellular volume fraction (i.e., myocardial reactive fibrosis ± edema) in patients with established cardiovascular disease or with cardiovascular risk factors.

It is now well-established that the location of fat deposition is much more closely linked to cardiovascular disease risk than the total quantity of fat (130). For instance, subcutaneous fat (located under the skin) accounts for 80–95% of total body fat but has relatively benign cardiometabolic consequences (130). When the finite limit of the expansion capacity of subcutaneous fat is reached, or in the presence of toxic exposures (e.g., smoking or chemotherapy), fat deposition occurs in ectopic “overflow” sites instead, including around the visceral organs (visceral), in skeletal muscle (intermuscular), in liver cells, or around the heart (epicardial) (130). Ectopic fat only accounts for 5–15% of total body fat but has much worse cardiometabolic consequences. For example, visceral and intermuscular fat deposition are independent predictors for cardiometabolic disease including hypertension, dyslipidemia, insulin resistance and atherogenesis and are predictive of cardiovascular mortality (131, 132). Furthermore, our group has shown that thigh intermuscular fat is a strong independent predictor of cardiorespiratory fitness level in male and female patients with HFpEF, breast cancer survivors, and females with cardiovascular risk factors (15, 133, 134). We have shown that visceral and intermuscular fat volume at the time of a breast cancer diagnosis predicts later cardiac events (135) and also significantly and rapidly increase with trastuzumab-based chemotherapy treatment (14).

**Figure 4:**
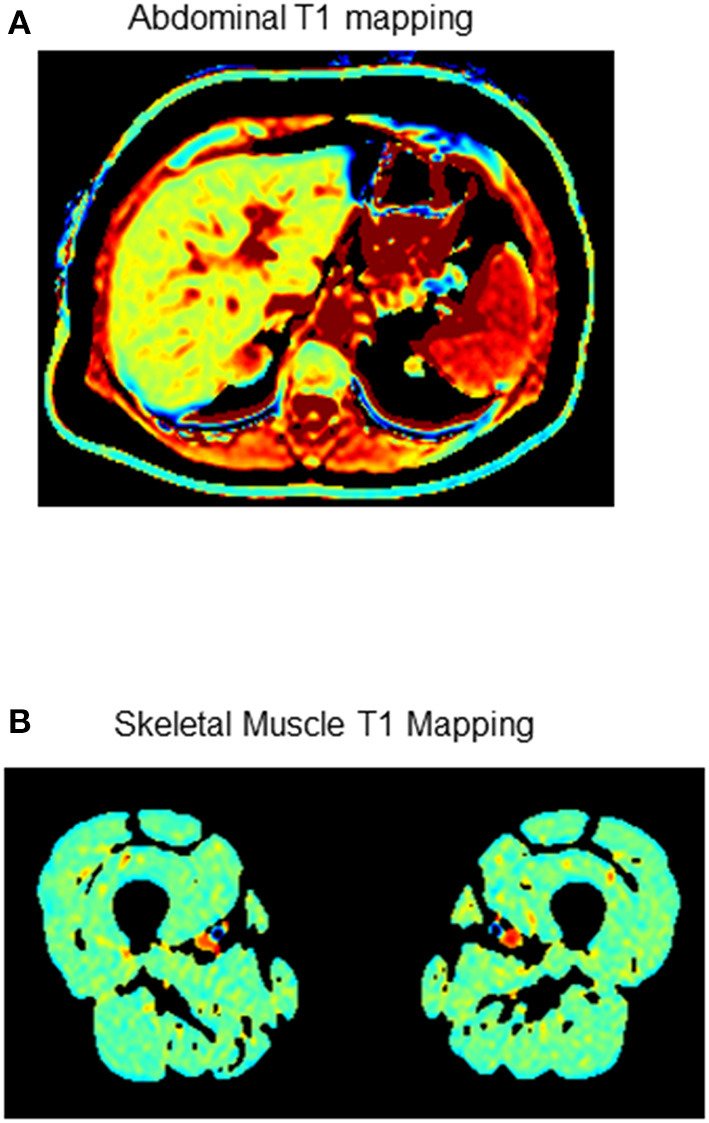
Examples of MRI T1 mapping sequences to assess the extra-cardiac effects of exercise training in patients with or at risk for cardiovascular disease. **(A)** Abdominal fat density **(B)** Skeletal muscle fat compartments. Dark signal = fat.

Imaging modalities used to quantify body composition in clinical practice and research include computed tomography, dual energy X-ray absorptiometry and MRI (Figure 4). MRI is uniquely suited to accurately and safely (without ionizing radiation) quantify fat in all of these locations as well as complementary information on cardiac structure and function in a single assessment.

Exercise training and caloric restriction (i.e., reduced daily calorie intake) are the primary interventions used to improve body composition. A meta-analysis comparing the effects of exercise training to caloric restriction in patients with obesity found that both interventions independently reduce visceral fat with a potential trend toward greater reduction with exercise (136). In patients with CAD undergoing cardiac rehabilitation, bioelectrical impedance-derived measures of visceral fat have been shown to improve, but with a greater magnitude of benefit with a greater volume of exercise training (137). Similarly, in a small study of patients with chronic heart failure, 5 months of cardiac rehabilitation also appears to reduce visceral adipose tissue (138). Data on the effects of exercise training in patients with hypertension is limited. However, one study of 156 hypertensive patients randomized to 12 months of exercise training or usual care found 30% reduction in visceral fat area on B-mode ultrasound compared to no effect with antihypertensive pharmacotherapy alone (139). Meta-analyses of the effect of exercise training on visceral fat in both overweight adults and patients with type 2 diabetes mellitus suggest that aerobic exercise training (but not resistance training) appears to provide the greatest benefits (140) (Table 3).

**Table 3:** Characteristics of the studies reporting on the extra-cardiac effects of exercise training.

**References**	**Study design** **(sample size)**	**Study population** **(age, %** **male)**	**Imaging type**	**Aerobic exercise prescription**	**Resistance exercise prescription**	**Exercise program duration**	**Relevant results**
**Obesity**							
Verheggen et al. (136)	Meta-analysis & Systematic Review of prospective studies (*n* = 4,815)	Obese patients Exercise group: *N* = 2404 (mean age range 21–73) Low calorie group: *N* = 2411 (mean age range 30–66, 33% male)	Computed tomography, MRI or DEXA	F: 1–7 days/week I: 40–90% VO_2peak_/HR_max_ T: 15–90 min	None	6–20 weeks	Both groups: ↓ weight & VAT Exercise relative to diet group: ↓ weight loss, trend ↑ VAT loss
**Coronary artery disease**							
Mirman et al. (137)	Prospective, two-arm trial (*n* = 715)	Clinically stable CAD Traditional cardiac rehab: *N* = 516 (median age 69, 74% male) Intensive cardiac rehab: *N* = 199 (median age 64, 78% male)	Bioelectrical impedance analysis	Traditional program: F: 2–3 days/week I: <70–85% HR_max_ T: 45–60 min Intensive program: F: 2 days/week I: <70–85% HR_max_ T: 240 min	F: 2–3 days/week I: not reported T: 15–30 min	Traditional: 8–12 weeks Intensive: 9 weeks	↓ weight, VAT & ↑ lean mass but greater effect in intensive program
**Heart failure**							
Takagawa et al. (138)	Prospective, single arm study (*n* = 19)	Chronic heart failure (any type)	Bioelectrical impedance analysis	F: 3–5 days/week I: anaerobic threshold−1 min T: 40–50 min	None	5 months	↓ weight & VAT ↔ lean mass
**Patients at risk for cardiovascular disease**							
Fang et al. (139)	RCT (*n* = 156)	Hypertensives (mean age 46 ± 8, 58% male)	B-mode ultrasound	F: 3 days/week I: 65% HR_max_ T: 60 min	None	12 months	↓ BMI and VAT only in exercise group
Sabag et al. (140)	Meta-analysis (*n* = 1,383)	Type 2 diabetes (mean age range 45–69, 37% male)	Computed tomography or MRI	F: 2–7 days/week I: 50–70% VO_2peak_, 60–90% HR_max_ T: 20–120 min	F: 3–5 days/week I: 40–80% 1-RM T: not reported	4 weeks−12 months	Aerobic training: ↓ VAT Resistance or combined training: ↔ VAT

*BMI, body mass index; CAD, coronary artery disease; DEXA, dual-energy x-ray absorptiometry; F, frequency; HR_max_, max heart rate; I, intensity; MRI, magnetic resonance imaging; RCTs, randomized control trials; T, time/duration; VAT, visceral adipose tissue; VO_2peak_, peak volume of oxygen consumption with exercise; 1-RM, 1-rep max*.

Our group is conducting a randomized-controlled trial of a multi-modal intervention including combined aerobic and resistance exercise training and dietary counseling (with no caloric restriction) on LV function in patients receiving cardiotoxic breast cancer treatment and includes quantification of ectopic fat as a secondary outcome measure (141). In a recent randomized controlled trial where exercise was performed shortly after the completion of breast cancer treatment, whole-body, and visceral fat volume were significantly reduced by 16-weeks of combined aerobic and resistance training (142). Therefore, another strategy to reduce cardiovascular risk in this population may be to perform an exercise intervention after treatment completion.

## Summary and Future Directions

Exercise training is a cornerstone treatment for patients with cardiovascular disease due its demonstrated impact on risk profile and clinical outcomes. Ultrasound or MR imaging for quantification of changes to the cardiac and extra-cardiac phenotype of patients, provide valuable whole-body information about the cardiovascular risk profile and have an excellent safety profile to allow for repeat testing.

The choice of imaging modality to evaluate changes in cardiovascular structure and function is an important consideration. In general, echocardiography is the most readily available modality and offers a comprehensive assessment of ventricular volumes, mass and function. It can also more easily be used to evaluate cardiac function in real time during exercise on a semi-recumbent bicycle. Cardiac MRI is considered the gold standard imaging test for ventricular volumes, mass and function due to high reproducibility. As mentioned previously, it also allows assessment of cardiac and extra cardiac tissue characterization which has increasingly been linked to prognosis in patients with or at risk for cardiovascular disease. Therefore, cardiac MRI should likely be used to study the effects of exercise training in higher risk patient groups when available.

There is strong evidence supporting a beneficial effect of exercise training, particularly >6 months duration, on cardiac function and remodeling in patients with CAD or heart failure (Table 4). Imaging studies on the effects of exercise training for vascular function in patients with or at risk for cardiovascular disease have shown either modest or no improvement in arterial stiffness. Furthermore, these studies are limited by the small number of participants, non-randomized design and lack of controlling for confounders.

**Table 4:** Overview of the exercise training effects of on cardiovascular imaging metrics in patients with or at risk for cardiovascular disease.

	**CAD**	**HFrEF**	**HFpEF**	**Hypertension**	**Diabetes mellitus**	**Cancer**
Systolic function	+	+	0	+/–	+/–	0
LV remodeling	+	+	0	+/–	+/–	0
Vascular function	+/–	0	0	0	0	?
Myocardial reactive fibrosis	?	?	?	?	?	?
Body composition	+	+/–	+/–	+/–	+	+/–

*+, beneficial effect*.

*+/–, possible beneficial effect, more studies needed*.

*0, no effect*.

*?, no or limited available data*.

*CAD, coronary artery disease; HFrEF, heart failure with reduced ejection fraction; HFpEF, heart failure with preserved ejection fraction; LV, left ventricular*.

Ectopic fat deposition, particularly, visceral and intermuscular fat are important indicators of cardiovascular risk that can be reduced by exercise training in numerous patient populations. MRI-acquired myocardial tissue characterization using T_1_ mapping sequences also provide incremental prognostic information however, the effect of exercise training on this parameter has not well-studied. MRI and/or ultrasound is well-suited for multiparametric evaluation of exercise training interventions and is easily translatable to the clinical setting to help guide patient management. Incorporation of MRI-derived lung water quantification could also help to identify cardiogenic causes of exercise intolerance and evaluate exercise training in patients with subclinical pulmonary edema (13, 143). Exercise cardiac reserve is another attractive approach to evaluating cardiogenic causes of exercise intolerance and detecting subclinical disease, and related studies are underway in the cancer setting (144, 145).

Targeted nutrition interventions can also positively impact cardiovascular health and should be further explored in combination with exercise interventions (146). Dietary patterns and specific nutrients can have independent, synergistic, or additive effects on reducing cardiovascular disease risk (147). Furthermore, nutrition interventions also have a therapeutic role for certain cardiovascular diseases (148). In addition to providing calories and macronutrients, specific nutrients can reduce oxidative damage, increase pro-inflammatory mediators and decrease anti-inflammatory mediators, while also promoting optimal body composition (e.g., decreasing ectopic fat, increase protein synthesis and reducing degradation) (148, 149).

Preclinical models of cardiovascular disease have been used extensively to study the cardioprotective effects of exercise training. Aerobic training has been shown to attenuate cardiac injury in animal models of myocardial infarction (150), ischemic heart failure (151), and preclinical disease (71, 152, 153). These models provide significant insight on the salient effects of exercise in cardiovascular disease and allow extensive histological and biochemical characterization of cardiovascular structure and function in a controlled setting. By comparison, resistance training protocols have been understudied in animal models of cardiovascular disease, in part due to a lack of standardized protocols. Nevertheless, beneficial cardiac and vascular adaptations with resistance training have been reported in small animal models of cardiovascular disease (154). Future preclinical work in exercise training requires refinement and standardization of training protocols to improve study comparisons and to better simulate exercise programs in the clinical setting (155). In conclusion, ultrasound and magnetic resonance-based imaging provides detailed information on the cardiac and extra-cardiac effects of exercise training. Imaging studies provide moderate or good evidence for exercise training to mitigate or improve cardiac remodeling and function in patients with or at risk for cardiovascular disease. Similar work on vascular function suggests a benefit in patients with CAD although these studies were non-randomized and included small numbers of patients. There is no clear benefit of exercise training in vascular function in patients with heart failure, hypertension or diabetes mellitus. There is little data on the effects of exercise training on replacement or reactive myocardial fibrosis. Future work on imaging studies of exercise training should also evaluate the complementary role of nutrition interventions given their importance in cardiac rehabilitation.

## Author Contributions

WA and DIP proposed the review subject matter and drafted the manuscript. All authors made critical revisions to the manuscript, contributed to the article, and approved the submitted version.

## Funding

MH was supported by the Faculty of Nursing Research Chair in aging and quality of life at the University of Alberta, EP was supported by a Tier 2 Canada Research Chair, and CP was supported by a Campus Alberta Innovates Program Chair in Nutrition, Food, and Health as well as a Canadian Institutes of Health Research new investigator award.

## Conflict of Interest

DIP reports consultant fees from Alnylam and Pfizer. The remaining authors declare that the research was conducted in the absence of any commercial or financial relationships that could be construed as a potential conflict of interest.

## Publisher's Note

All claims expressed in this article are solely those of the authors and do not necessarily represent those of their affiliated organizations, or those of the publisher, the editors and the reviewers. Any product that may be evaluated in this article, or claim that may be made by its manufacturer, is not guaranteed or endorsed by the publisher.
